# Obesity Does Not Increase Mortality after Emergency Surgery

**DOI:** 10.1155/2014/492127

**Published:** 2014-02-16

**Authors:** Paula Ferrada, Rahul J. Anand, Ajai Malhotra, Michel Aboutanos

**Affiliations:** ^1^Trauma, Critical Care and Emergency Surgery, Virginia Commonwealth University, West Hospital, 15th Floor East, 1200 E. Broad Street, P.O. Box 980454, Richmond, VA 23298, USA; ^2^Department of Surgery, Virginia Commonwealth University, Richmond, VA 23298, USA

## Abstract

*Objective.* The aim of this study is to evaluate the impact of obesity on patient outcomes after emergency surgery. *Methods.* A list of all patients undergoing emergent general surgical procedures during the 12 months ending in July 2012 was obtained from the operating room log. A chart review was performed to obtain the following data: patient characteristics (age, gender, BMI, and preexisting comorbidities), indication for surgery, and outcomes (pulmonary embolus (PE), deep venous thrombosis (DVT), respiratory failure, ICU admission, wound infection, pneumonia, and mortality). Obesity was defined as a BMI over 25. Comparisons of outcomes between obese and nonobese patients were evaluated using Fischer's exact test. Predictors of mortality were evaluated using logistic regression. *Results.* 341 patients were identified during the study period. 202 (59%) were obese. Both groups were similar in age (48 for obese versus 47 for nonobese, *P* = 0.42). Obese patients had an increased incidence of diabetes, (27% versus 7%, *P* < 0.05), hypertension (52% versus 34%, *P* < 0.05), and sleep apnea (0% versus 5%, *P* < 0.05). There was a statistically significant increased incidence of postoperative wound infection (obese 9.9% versus nonobese 4.3%, *P* < 0.05) and ICU admission (obese 58% versus nonobese 42%, *P* = 0.01) among the obese patients. Obesity alone was not shown to be a significant risk factor for mortality. *Conclusions.* A higher BMI is not an independent predictor of mortality after emergency surgery. Obese patients are at a higher risk of developing wound infections and requiring ICU admission after emergent general surgical procedure.

## 1. Introduction

Obesity is a growing health concern both nationally and globally [1, 2]. Obese individuals are at increased risk of having serious health problems, including hypertension, dyslipidemia, coronary artery disease, respiratory problems, and diabetes mellitus to name a few [3]. Body mass index (BMI) is the standard measure of obesity [4]. Increased BMI has been reported as a risk factor for several causes of death, including ischemic heart disease and stroke [5].

Studies evaluating the outcome of obese patients admitted to the intensive care unit (ICU) [6, 7] have shown contradictory results regarding the impact of BMI in morbidity and mortality [8, 9]. Moreover, there is a paucity of data regarding obese patients undergoing emergency surgery.

This is an initial pilot study to evaluate if patients with a higher BMI have more complications and increased mortality after emergency surgery in order to create awareness and potentially allocate resources for further research and stratification of obesity in this specific patient population. The groups were divided between obese/overweight (patients had a BMI higher than 25) and nonobese to be able to include more patients in the obese group and evaluate the impact of BMI in acute care surgery patients. We will refer to this as the obese group.

## 2. Methods

The study was approved by the Institutional Review Board at the Virginia Commonwealth University. A list of all patients undergoing emergency general surgical procedures (posted as less than 2 hours to the OR) during the 12 months ending in July 2012 was obtained from the operating room log. A chart review was performed to obtain data including patient age, gender, BMI, and presence of comorbidities including previous deep venous thrombosis (DVT), pulmonary embolism (PE), coronary artery disease (CAD), peripheral artery disease (PVD), chronic obstructive pulmonary disease (COPD), hypertension (HTN), diabetes, and obstructive sleep apnea (OSA). Data regarding patient outcomes was also collected including development of respiratory failure, PE, DVT, wound infection, and pneumonia.

Patients who underwent cardiac, trauma, and vascular procedures were removed from the database in an effort to evaluate patients who underwent emergency acute care general surgery only. All cases were listed as emergency. Of the entire group, 70% of the patients were in the emergency room before the surgical procedure took place. Surgical procedures were coded as appendectomy (*n* = 28), small bowel obstruction (*n* = 35), necrotizing soft tissue infection (*n* = 53), abscess (*n* = 62), free air (*n* = 32), colitis (*n* = 15), peritonitis (*n* = 18), bowel ischemia (*n* = 28), bowel perforation (*n* = 11), GI bleeding (*n* = 6), perforated diverticulitis (*n* = 16), leak (*n* = 8), and incarcerated hernia (*n* = 29).

The groups were divided between obese/overweight (patients had a BMI higher than 25) and nonobese to be able to include more patients in the obese group and evaluate the impact of BMI in acute care surgery patients. In other words, to prevent ourselves from missing BMI related complications, we wanted to be inclusive in our criteria.

Comparisons between groups were evaluated using Fischer's exact test. Predictors of mortality were evaluated using logistic regression. All analyses were performed using SAS 9.3 software (Cary, NC, USA). Statistical difference was defined as a *P* value less than 0.05 (*P* < 0.05).

## 3. Results

341 patients were identified during the study period. 202 (59%) patients had a BMI higher than 25 kg/m^2^. In the obese/overweight group, 112 patients (55.4%) had a BMI higher than 30 kg/m^2^, meeting the CDC criteria for obesity. The average BMI of the obese/overweight group was 38.8 kg/m^2^ with a range of 30.5 to 70.41, and the nonobese group had an average BMI of 23.2 kg/m^2^ with a range from 16.5 to 29.1.

Both groups were similar in age (47.1 versus 48.6, *P* = 0.42). Obese/overweight patients, compared to their nonobese counterparts, had an increased incidence of diabetes (27% versus 7%, *P* < 0.05), hypertension (52% versus 34%, *P* < 0.05), and OSA (0% versus 5%, *P* < 0.05). See Table 1.

Although obese patients had an increased incidence of some postoperative complications, there was no statistically significant difference in the occurrence of postoperative PE (obese 1.5% versus nonobese 2.2%, *P* = 0.69), postoperative DVT (4.5% versus 2.2%, *P* = 0.37), postoperative pneumonia (3.5% versus 2.2%, *P* = 0.74), or respiratory failure (16.8% versus 12.2%, *P* = 0.28). There was a statistically significant increased incidence of postoperative wound infection (9.9% versus 4.3%, *P* < 0.05) and ICU admission (58% versus 42%, *P* < 0.05) among the obese patients. See Table 2. All of the ICU admissions were to the surgical intensive care unit with the exception of 2 patients in the nonobese group. The database does not delineate specific reasons for ICU admission. Mortality was not higher in the obese group when compared with the nonobese group (15.8% versus 13.6%, *P* = 0.60).

Logistic regression was used using each variable to predict mortality. The variables included in the analysis were diabetes, hypertension, deep venous thrombosis, pulmonary emboli, obstructive sleep apnea, peripheral vascular disease, coronary artery disease, and BMI >25. Obesity alone was not shown to be a risk factor for mortality. Patients with CAD and PVD were shown to have a significantly higher mortality after emergency surgery procedures (PVD; odds ratio = 3.674, CI = 1.131–11.933, *P* < 0.05; CAD; odds ratio = 2.850, CI = 1.390–5.840, *P* < 0.05) regardless of BMI. The population of patients with PVD and CAD was too small to compare obese and nonobese counterparts (PVD nonobese *n* = 9, PVD obese *n* = 6; CAD nonobese *n* = 23, CAD obese *n* = 34).

## 4. Discussion

Obesity is a growing health care problem, especially in the United States [5, 10]. This is problematic for the surgical community since obesity has been shown to increase the risk of perioperative complications [11, 12]. Obese patients require an aggressive preventive strategy to decrease the rate of adverse events [13, 14]. Patients undergoing emergency surgery are a group of patients who demand special care, due to the fact that mortality in this group is eight times that of patients undergoing elective procedures [15]. In the present study, however, obese patients undergoing emergency surgery had a higher incidence of comorbidities but were not shown to have a higher mortality rate.

The previous literature has agreed with our findings that obesity is not a risk factor for increased mortality [16]. One explanation for the lack of difference could be that obese patients are subject to heightened awareness on the part of the care teams for potential complications. However, in the current study, obese patients still developed more wound infections than nonobese counterparts.

A recent systematic review supports the association of obesity with higher mortality in trauma patients [8]. In this paper the authors highlighted the complexity of the postoperative care of obese patients resulting in longer ICU stays and increased complications. Similarly, in this study, obese patients had a higher rate of ICU admission, requiring more resources to get this population healthy and safely discharged.

## 5. Conclusions

In summary, in our center obese patients requiring emergency surgery did not have increased mortality. Higher mortality was associated with CAD and PVD. Attention needs to be given to factors to decrease the cardiac complications as well as postoperative soft tissue infections.

## Figures and Tables

**Table 1:** Demographics and comorbidities.

Study groups	Age	% of males	DM	HTN	DVT	PE	COPD	OSA	PVD	CAD
Nonobese (*n* = 139)	47.1	65.47	10	47	3	1	10	0	9	23
Obese (*n* = 202)	46.8	49.5	54	105	10	6	15	11	6	34

DM: diabetes mellitus, HTN: hypertension, DVT: deep venous thrombosis, PE: pulmonary embolism, COPD: chronic obstructive pulmonary disease, OSA: obstructive sleep apnea, PVD: peripheral vascular disease, and CAD: coronary artery disease.

**Table 2:** Postoperative comorbidities.

Study groups	Mortality	ICU	Pneumonia	Wound infection	Postoperative PE	Postoperative DVT	RF
Nonobese (*n* = 139)	15.80%	42%	2.20%	4.30%	2.20%	2.20%	12.20%
Obese (*n* = 202)	13.60%	58%	3.50%	9.95%	1.50%	4.50%	16.80%

PE: pulmonary embolism, DVT: deep venous thrombosis, and RF: respiratory failure.
